# Investigation of the Chemiluminescent Reaction of a Fluorinated Analog of Marine Coelenterazine

**DOI:** 10.3390/ma17040868

**Published:** 2024-02-13

**Authors:** Carla M. Magalhães, Joaquim C. G. Esteves da Silva, Luís Pinto da Silva

**Affiliations:** Centro de Investigação em Química (CIQUP), Instituto de Ciências Moleculares (IMS), Departamento de Geociências, Ambiente e Ordenamento do Território, Faculdade de Ciências, Universidade do Porto, Rua do Campo Alegre s/n, 4169-007 Porto, Portugal; up201201533@fc.up.pt (C.M.M.); jcsilva@fc.up.pt (J.C.G.E.d.S.)

**Keywords:** chemiluminescence, bioluminescence, coelenterazine, chemiexcitation

## Abstract

Bioluminescence (BL) and chemiluminescence (CL) are remarkable processes in which light is emitted due to (bio)chemical reactions. These reactions have attracted significant attention for various applications, such as biosensing, bioimaging, and biomedicine. Some of the most relevant and well-studied BL/CL systems are that of marine imidazopyrazine-based compounds, among which Coelenterazine is a prime example. Understanding the mechanisms behind efficient chemiexcitation is essential for the optimization and development of practical applications for these systems. Here, the CL of a fluorinated Coelenterazine analog was studied using experimental and theoretical approaches to obtain insight into these processes. Experimental analysis revealed that CL is more efficient under basic conditions than under acidic ones, which could be attributed to the higher relative chemiexcitation efficiency of an anionic dioxetanone intermediate over a corresponding neutral species. However, theoretical calculations indicated that the reactions of both species are similarly associated with both electron and charge transfer processes, which are typically used to explain efficiency chemiexcitation. So, neither process appears to be able to explain the relative chemiexcitation efficiencies observed. In conclusion, this study provides further insight into the mechanisms behind the chemiexcitation of imidazopyrazinone-based systems.

## 1. Introduction

**Scheme 1:**
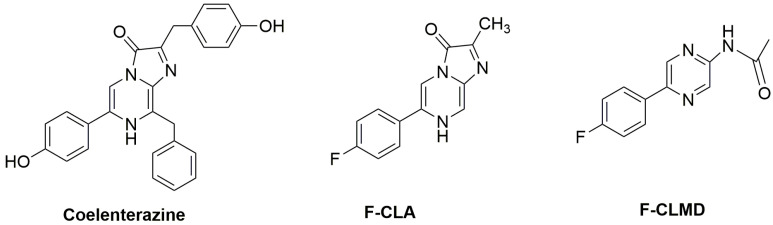
Schematic depiction of native Coelenterazine, F-CLA, and F-CLMD.

**Scheme 2:**
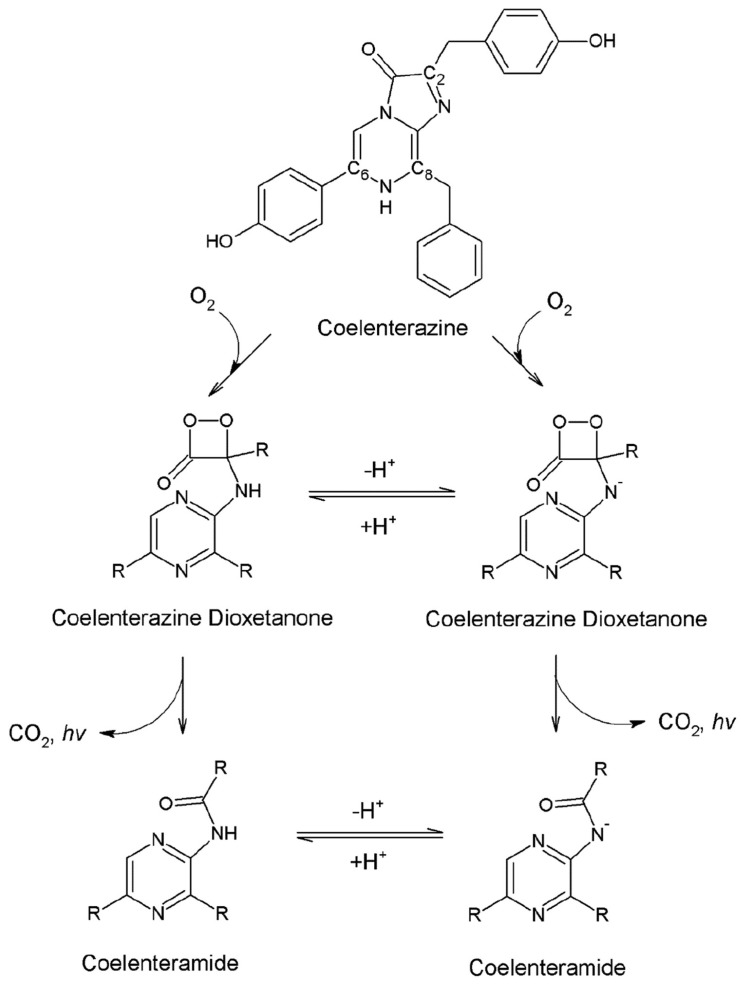
General schematic depiction of the CL/BL reaction mechanism of Coelenterazine, reproduced here with authorization from [53].

Bioluminescence (BL) and chemiluminescence (CL) represent processes in which light is emitted because of (bio)chemical reactions [1,2]. These systems have gathered considerable attention within the research community, with a focus on their diverse applications in sensing [3], real-time imaging [4,5], hygiene control [6], mapping pollution in ecosystems [7,8], and even in drug discovery [9] and development [10,11]. The heightened interest in BL and CL systems stems, in part, from their association with a reduced probability of autofluorescence originating from background signal, as they do not necessitate photoexcitation [12,13]. Consequently, luminescent signals are generated with minimal background noise [11]. BL is widespread in nature and can be found in living organisms as diverse as fireflies, jellyfish, and fungi [14,15,16]. However, about 80% of all luminescent organisms are present in oceans [17]. Imidazopyrazinone-based BL substrates are among the most common substrates, with marine Coelenterazine being one of the most well-known and studied compounds (Scheme 1) [18,19,20,21,22,23,24]. Coelenterazine is an interesting compound as it is capable of both CL (when triggered by reactive oxygen species or in aprotic solvents, such as Dimethyl sulfoxide, DMSO, and Dimethylformamide (DMF)) [25,26,27,28], and BL (when in the presence of photoproteins or luciferase enzymes) [28,29,30]. Despite these differences, BL/CL reactions of Coelenterazine follow the same general mechanism (Scheme 2), which is initiated by oxygenation of the imidazopyrazinone core [31,32], with the formation of a high-energy peroxide intermediate. This latter compound is highly unstable and undergoes thermolysis almost instantly. During this reaction, the reacting molecules can cross to singlet excited states, which generates the chemiexcited light-emitter Coelenteramide [33,34,35,36].

Among different research efforts made by the community regarding this type of system is the development of new Coelenterazine analogs with enhanced properties, such as brighter light-emission and a longer emission half-life, toward their optimized application [37,38,39,40]. It should be highlighted that for the rational development of new Coelenterazine analogs, a precise understanding of the different steps involved in their CL and BL reaction mechanisms in needed. However, there are still some issues that require clarification. For instance, the mechanism behind singlet chemiexcitation is not yet fully understood, nor is the ionization state of the dioxetanone intermediate that is associated with efficient chemiexcitation agreed upon.

The first mechanism used to rationalize efficient singlet chemiexcitation was Chemically Induced Electron-Exchange Luminescence, or CIEEL [40]. It is based on electron transfer from an electron-donating group to the peroxide group, followed by back electron transfer, which generates the chemiexcited light-emitter [40,41]. This mechanism has been used to rationalize efficient CL/BL in diverse systems, such as in fireflies [42]. CIEEL was indeed shown to be operative in the efficient peroxyoxalate system [41]. However, re-evaluations of other previously thought efficient model CIEEL systems revealed them to be less efficient than expected [43,44].

Other researchers have tried to re-formulate the CIEEL mechanism in terms of more gradual charge transfer and back charge transfer between the electron-rich group and peroxide moiety, usually involving anionic species. This alternative version was termed Charged Transfer-Initiated Luminescence, or CTIL [45,46]. However, it should be noted that the requirements of either electron-transfer-based or charge-transfer-based mechanisms are similar, and that charge transfer encompasses electron transfer [41,47].

Specifically regarding imidazopyrazinone-based systems, Saito and co-workers claimed that CIEEL/CTIL mechanisms are not applicable for explaining the efficient chemiexcitation yield in aqueorin BL [48]. They also stated that they were not able to validate the expectations that electron-donating groups could enhance the singlet chemiexcitation yield [48]. In turn, Hirano et al. indicated that efficient chemiexcitation involving an imidazopyrazinone analog is explained by considering a neutral dioxetanone intermediate [49]. This is relevant as theoretical studies have shown that the chemiexcitation of neutral dioxetanone species tends not to follow the CIEEL/CTIL mechanism [23,50,51]. Furthermore, a lack of correlation between charge/electron transfer and efficient singlet chemiexcitation for imidazopyrazinone systems has been observed [33,50,51,52].

Given this, it is important to further clarify the CL and BL mechanism of imidazopyrazinone-based systems. To that end, we investigated the CL reaction of a fluorinated Coelenterazine analog (F-CLA, Scheme 1). To try to understand the potential effects of charge/electron transfer, we replaced the electron-donating hydroxyl group of the phenyl moiety of native Coelenterazine (Scheme 1) with electron-withdrawing fluorine [53,54]. This analog also replaced the benzyl and *p*-cresol moieties of Coelenterazine with a hydrogen atom and a methyl group. This was performed to decrease the functionalization degree to facilitate analysis of the results by reducing the potential effects exerted by other moieties present in Coelenterazine. With this study, we aimed to provide further insight into the CL reaction of Coelenterazine, which can help to develop future analogs with enhanced properties.

## 2. Materials and Methods

The synthesis of F-CLA followed a general synthetic pathway, starting with a Suzuki–Miyaura cross-coupling reaction between 5-bromopyrazin-2-amine and the corresponding organoborane. This step yielded an intermediate known as Coelenteramine, an aminopyrazine-based compound. More specifically, a solution containing methyl glyoxal and 3-bromo-5-(4-fluorophenyl)pyrazin-2-amine (a fluorinated Coelenteramine analog, F-CLM), dissolved in ethanol, was deoxygenated with N_2_ [25]. It should be noted that the Coelenteramine analog was prepared as described in [55]. Following deoxygenation, the mixture was cooled to 0 °C, treated with hydrochloric acid, and stirred until it reached room temperature. The solution was then stirred at 70 °C for 2.5 h and left at room temperature overnight. After concentration under reduced pressure, a brown oil was obtained, redissolved in minimal ethyl acetate, and subsequently precipitated with diethyl ether. The resulting product, F-CLA, was vacuum-dried, presenting as a solid ochre. The fluorinated Coelenteramide analog, F-CLMD (Scheme 1), was obtained by the N-acetylation of F-CLM using pyridine as a base to avoid the formation of a disubstituted subproduct, as depicted in [56]. The structural confirmation of both F-CLA and F-CLMD was conducted through NMR and FT-MS analysis, as demonstrated in [25] and [56], respectively.

CL kinetic measurements were performed using a homemade luminometer equipped with a Hamamatsu HC135-01 photomultiplier tube in a setup that included a sample holder, an automatic burette, and a PC for data acquisition. This homemade setup has been used before in the study of both CL and BL reactions [21,24,25]. The reactions were performed at least in sextuplicate at room temperature in DMSO, to which either sodium acetate buffer pH 5.2 or sodium hydroxide solution was added (to a common final concentration of 0.1 M). Final volumes of 500 μL and an F-CLA concentration of 5 μM were used. Light was integrated and recorded at 0.1 s intervals. CL was triggered spontaneously by the addition of DMSO mixtures to F-CLA.

The fluorescence of F-CLMD was monitored using a Horiba Jovin Fluoromax 4 spectrofluorimeter, with an integration time of 0.1 s and slit widths of 5 nm for both the excitation emission monochromators. UV-Vis spectra for F-CLMD were obtained using a UV-3100PC spectrophotometer. Both types of spectra were obtained in 2 mL solutions of DMSO, with either sodium acetate buffer pH 5.2 or sodium hydroxide (final concentration of 0.1 M for both buffer and base), with a final compound concentration of 5 μM.

Theoretical calculations were performed using the Gaussian 09 program package [57]. *S_0_* geometry optimization and frequency calculations of the transition state of neutral and anionic species of F-CLM dioxetanone were conduced with the ωB97XD density functional [58,59] and 6-31G(d,p) basis set. An open-shell approach was used, along with broken-symmetry technology, to make an initial guess for a biradical [60]. The transition states were located considering initial guesses based on previous studies on this type of system [24,33,50,51,52]. ωB97XD was chosen as long-range-corrected hybrid exchange-correlation functionals, including this one, are known to provide good results for this type of system [25,34,51,52,53]. *S_0_* single-point energy calculations were performed at the ωB97XD/6-31+G(d,p) level of theory, on top of *S_0_* ωB97XD/6-31G(d,p) geometries. Single-point calculations were performed in implicit DMSO, whereas geometry optimizations were performed in the gas phase. Implicit solvation was considered using a polarizable continuum model (IEFPCM). This calculation framework has been used previously in different studies [23,24,33,50,51,52,60,61].

## 3. Results and Discussion

The aim of this study was to obtain more insight into the CL and BL mechanism of imidazopyrazinone-based compounds, with a focus on the chemiexcitation step and the reason that controls its efficiency. To that end, we studied F-CLA, a Coelenterazine analog that possesses an electron-withdrawing fluorine instead of the native electron-donating hydroxyl group present in the native compound. With this difference, we intended to obtain more information on the potential role of electron/charge transfer in chemiexcitation, as postulated by CIEEL and CTIL mechanisms [40,41,45,46]. This study was performed in DMSO, a polar aprotic solvent in which Coelenterazine and other imidazopyrazinones readily chemiluminesce upon mixing [24,48,49,50,62]. It should be noted that the CL reactions of imidazopyrazinones have been typically considered a common and reliable model for their BL reaction [24,48,49,50,62,63,64,65], which means that the results of this CL-focused study should also be of interest and applicable to imidazopyrazinone-based BL systems.

Measurements were performed in solutions to which sodium acetate buffer solution (pH 5.2) or sodium hydroxide was added. This was performed to achieve acidic and basic conditions in order to consider the neutral-anionic chemical equilibria of imidazopyrazine-based dioxetanones and their impact on the chemiexcitation step (Scheme 2) [23,51]. Namely, the chemiexcitation of neutral dioxetanones is expected to occur via non-CIEEL/CTIL pathways, whereas anionic dioxetanones are associated with CIEEL/CTIL mechanisms [23,50,51,66,67]. To reduce potential interfering factors, both buffer and base were added at the same concentration and with the same counter cation (sodium ion).

**Figure 1:**
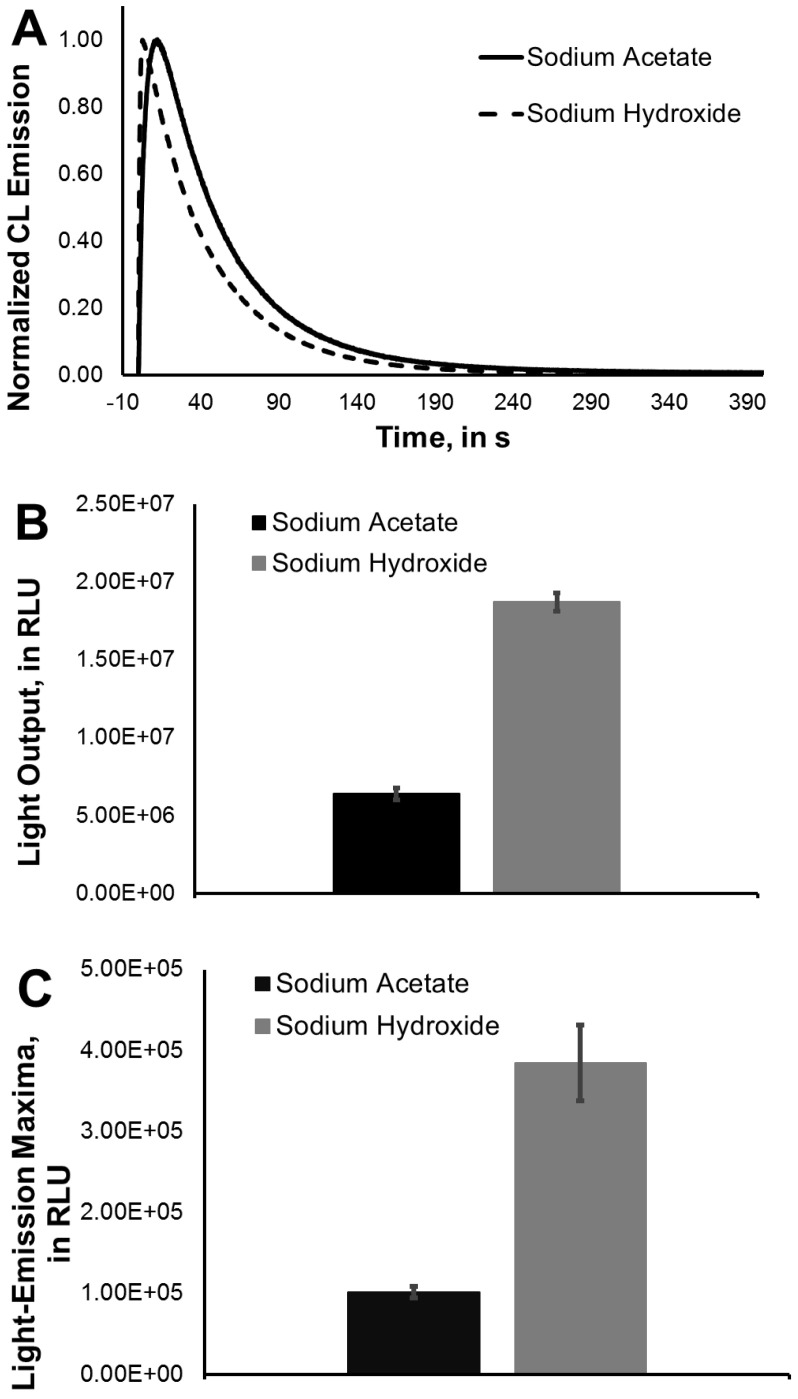
(**A**): Normalized CL kinetic profile for F-CLA in either DMSO/sodium acetate buffer pH 5.2 or in DMSO/sodium hydroxide. (**B**): Total light output (area) measured for F-CLA in either DMSO/sodium acetate buffer pH 5.2 or in DMSO/sodium hydroxide. (**C**): Light-emission maxima measured for F-CLA in either DMSO/sodium acetate buffer pH 5.2 or in DMSO/sodium hydroxide.

The CL kinetic profiles were then obtained for F-CLA in both DMSO/sodium acetate buffer and DMSO/sodium hydroxide (Figure 1A). The obtained profiles show a typical flash-profile for this type of system, with a quick burst of light, followed by decay to basal levels within the first 200 s of the reaction [21,24,25,50,55]. Although the kinetic profiles appear to be qualitatively identical, we also observed that the emission lifetime of F-CLA appeared to be longer in acidic conditions. The measured CL half-life (in s) is 46.6 ± 1.0 s and 28.9 ± 3.6 s in acidic and basic conditions, respectively. This shorter emission lifetime in basic media is consistent with previous reports of halogenated imidazopyrazinone-based CL reactions [68,69].

**Figure 2:**
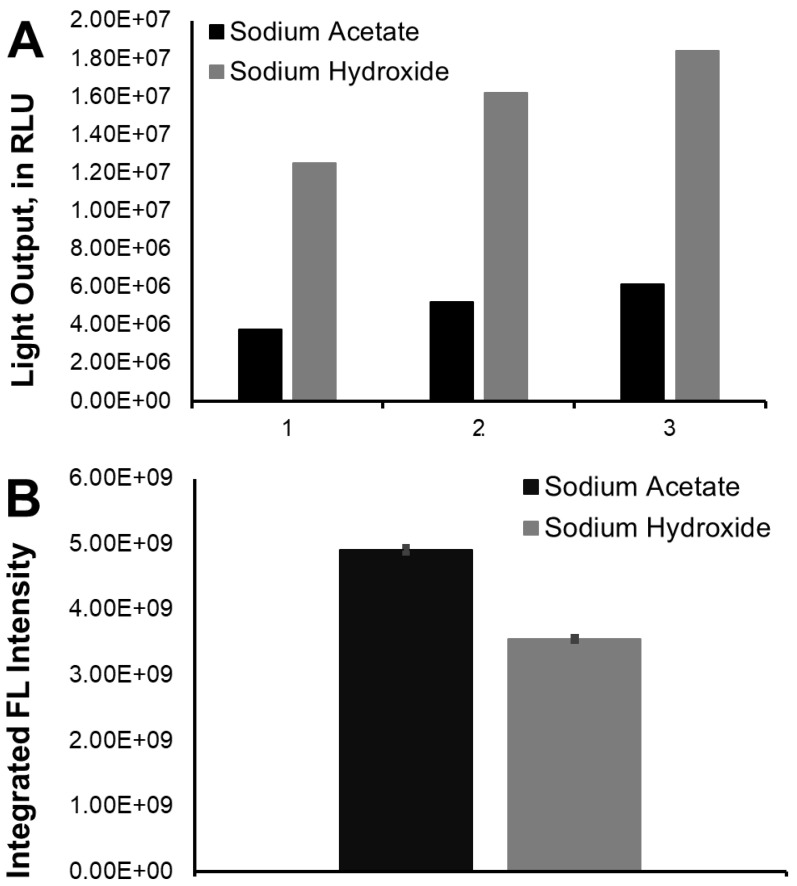
(**A**): Total light output (area) measured for F-CLA in either DMSO/sodium acetate buffer pH 5.2 or in DMSO/sodium hydroxide. The number 1 accounts for the first 50 s of the reaction, 2 accounts for the first 100 s, and 3 accounts for the first 300 s. (**B**): Integrated fluorescence (FL) intensity (area of the spectrum) measured for F-CLMD in either DMSO/sodium acetate buffer pH 5.2 or in DMSO/sodium hydroxide, with excitation at 300 nm.

To evaluate whether these different emission lifetimes correlated with higher/lower light production, a quantitative analysis was performed by measuring both the calculated area of light emission (in RLU, Figure 1B) and the light-emission intensity maxima (in RLU, Figure 1C). The area corresponds to emitted light as a function of time and can be considered as a measure of total light output, and so, is indicative of CL quantum yield [24]. The area was calculated between times of 0 and 600 s. We observed that both the total light output and the light-emission intensity maxima were significantly higher and to similar degrees in basic conditions compared to acidic ones. The area of light emission was also calculated at smaller time intervals (0–50 s, 0–100 s, and 0–300 s, in Figure 2A), to verify whether the observed relative differences were consistent throughout the entire reaction. In fact, there was no difference in relative light output during the CL reactions in acidic and basic conditions at these different times. This means that the lower emission lifetime in basic conditions is not associated with less efficient light production.

Considering these results, it was important to understand why F-CLA is more efficient in the generation of light in basic conditions than in acidic conditions. It should be noted that both BL and CL quantum yields are controlled by three different parameters [1,2]. This includes the yield of the *S_0_* chemical reaction, the singlet chemiexcitation yield, and the fluorescence quantum yield of the light-emitter in this type of system, Coelenteramide. Thus, the observed pH-dependency could potentially originate from one parameter or from a combination of effects regarding two or three parameters. 

In these experiments, we measured light-emission until and well after reaching basal levels in both acidic and basic conditions (with measurements up to 600 s). We observed similar kinetic profiles with the addition of either buffer solution or sodium hydroxide (Figure 1A), indicating similar completions of the reaction and levels of F-CLA consumption. In fact, Figure 1A indicates that basal levels are reached at similar reaction times for both pH conditions. Given this, we did not expect the differences in relative light output to result from pH-induced differences in the *S_0_* chemical reaction yield.

Consequently, both the chemiexcitation and fluorescence quantum yields remained under consideration. Both are possible explanations for the observed pH-dependent light-emission efficiency. Namely, chemiexcitation involves the dioxetanone intermediate, which presents a neutral–anionic chemical equilibrium (Scheme 2) [23,51]. The Coelenteramide light-emitter also presents a neutral–anionic equilibrium, which involves the amide group [70]. Direct evaluation of the chemiexcitation step is experimentally difficult; therefore, it should be assessed indirectly by the exclusion of other parameters. Thus, combined with our opinion that the *S_0_* chemical reaction was complete (or at least the yield was identical at both pH ranges), we shifted our attention to the analysis of the fluorescence-related parameter.

**Figure 3:**
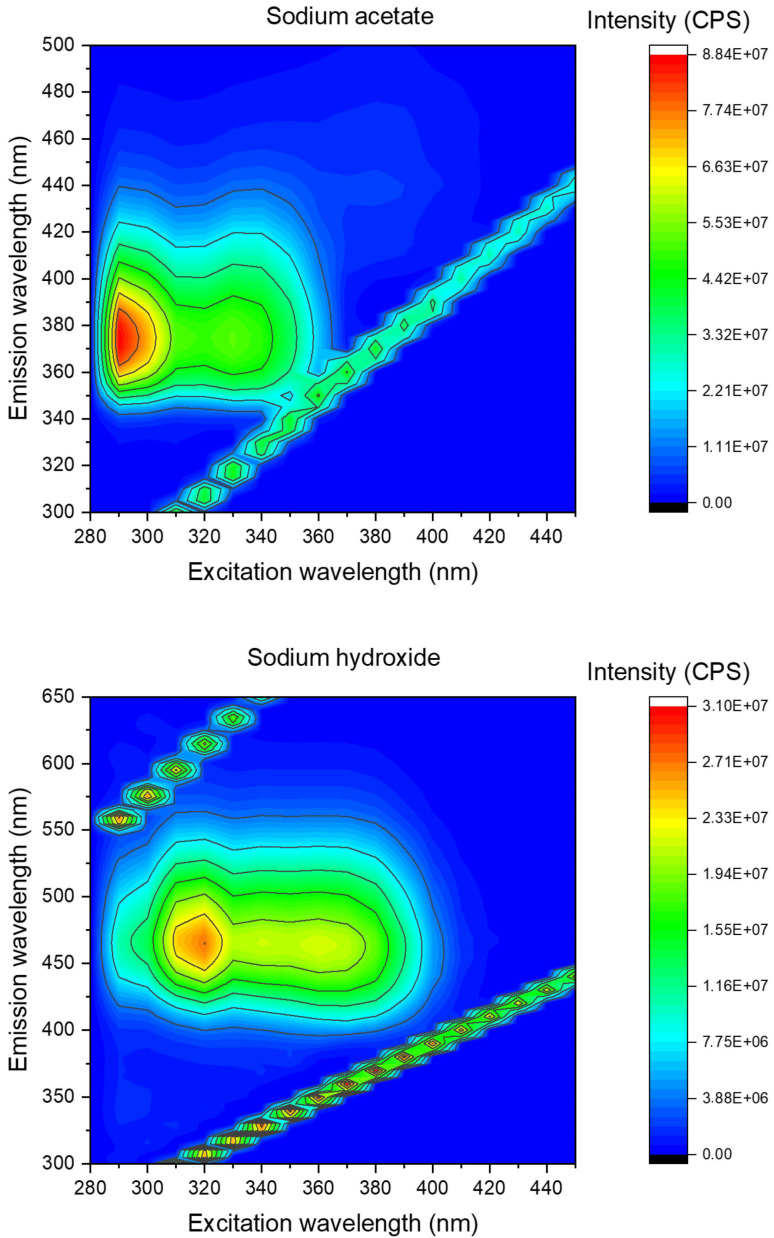
Two-dimensional excitation–emission contour plot for F-CLMD, measured in either DMSO/acetate buffer pH 5.2 (top) or in DMSO/sodium hydroxide (bottom).

To that end, we synthesized the corresponding F-CLMD, the expected light-emitter of the CL reaction, [56] and measured its fluorescence in both DMSO/sodium acetate buffer and DMSO/sodium hydroxide. The resulting 2D excitation–emission contour plots are provided in Figure 3. The obtained results are consistent with the existence of the neutral–anionic amide equilibrium for F-CLMD (Scheme 2) [70]. More specifically, in DMSO/sodium acetate buffer, F-CLMD presents an emission wavelength maximum at ~375 nm. This is in line with the fluorescence of this compound as measured previously in DMSO only [56]. However, under basic conditions, we observed a red-shift in emission to ~460 nm. This increase in emission wavelength maxima, with changes in pH, can be attributed to the deprotonation of the amide groups of Coelenteramide compounds [27,70]. So, the fluorescence of F-CLMD appears to be pH-dependent and is a possible explanation for the pH-dependent CL of F-CLA.

The fluorescence quantum yield of a fluorophore is considered as the ratio of absorbed photons to photons emitted via fluorescence. In essence, it is the probability of excited states being deactivated by fluorescence instead of nonradiative pathways. Fluorescence quantum yields can be measured using comparative methods, in which two fluorophores with identical absorbance at the excitation wavelength are assumed to absorb the same number of photons. Given this, the fluorescence quantum yield ratio of the two fluorophores is obtained by considering the ratio of the integrated fluorescence intensities of the compounds.

Considering this, we measured the integrated fluorescence intensities of F-CLMD at the same concentration (5 μM) in both DMSO/sodium acetate and DMSO/sodium hydroxide when using the same excitation wavelength (300 nm). These measurements were performed as a simple approximation for evaluating the fluorescence quantum yield ratio of F-CLMD at acidic and basic pH. That is, the difference in fluorescence quantum yields between neutral and anionic amide species of this light-emitter. With this approach, we intended to assess whether the higher CL light output measured at basic pH could be attributed to the higher fluorescence quantum yield of the light-emitter (anionic amide F-CLMD) associated with higher pH values.

Contrary to this hypothesis, we found that the integrated fluorescence intensity was relevantly higher at acidic pH than at basic pH (Figure 2B). This result points to the fluorescence quantum yield of neutral F-CLMD being higher than that of the corresponding anionic species. It should be noted that the absorbance measured at 300 nm, in either DMSO/sodium acetate or DMSO/sodium hydroxide, was not the same. In fact, it was lower in the former mixture than in the latter mixture (0.06 versus 0.14). Thus, it is expected that F-CLMD absorbs more photons at basic pH than in acidic conditions. However, despite this, the integrated fluorescence intensities were lower in basic conditions, as mentioned before (Figure 2B). Considering the notion that fluorescence quantum yield is the ratio of absorbed photons to photons emitted via fluorescence, these results indicate that this parameter is lower for anionic F-CLMD than for the corresponding neutral species. This is consistent with the 2D excitation–emission contour plots, in which the overall fluorescence intensities were lower under basic conditions than in an acidic buffer (Figure 3).

Having reached this conclusion, the next logical step was to attribute the higher CL light output at basic pH to a higher chemiexcitation yield in those conditions, considering the exclusion of other parameters responsible for the CL quantum yield. In fact, the difference in chemiexcitation yields between the pH ranges should be particularly relevant to allow higher overall yields under basic pH, despite the relatively lower fluorescence quantum yield of the light-emitter at those pH conditions. Given that these expected differences in chemiexcitation yields occur under acidic/basic conditions, they can be attributed to the known neutral–anionic chemical equilibrium of dioxetanone intermediate (Scheme 2). That is, it is the decomposition of this intermediate that leads to chemiexcitation, with diverse studies indicating that the two species (neutral/anionic) are associated with different chemiexcitation pathways [23,33,50,51,52,66,67].

It should be noted that these results are particularly interesting because they are different from what has been reported in previous studies involving Coelenterazine and other imidazopyrazinones. That is, pH-dependency has already been previously reported for this type of system, but contrary to what is observed here, higher light outputs have been measured under acidic conditions [49,50,52,62,68]. Moreover, these higher yields have been attributed to chemiexcitation with neutral dioxetanones [49,50,52,62,68], whereas, in this study, it appears that anionic dioxetanone leads to higher chemiexcitation yields and, consequently, higher light outputs.

Given this, understanding the reason behind the differences observed between the results obtained in this study and in previously published works might help us obtain further insight into the mechanisms behind the chemiexcitation efficiencies of imidazopyrazinone-based systems. To that end, we then performed DFT-based calculations to evaluate the chemiexcitation mechanisms of F-CLA. More specifically, we optimized the *S_0_* geometry of the TS structures of the thermolysis reactions of both neutral and anionic F-CLA dioxetanone species at the ωB97XD/6-31G(d,p) level of theory. Subsequent single-point energy calculations were then performed at the ωB97XD/6-31+G(d,p) level of theory in implicit DMSO to assess the electron and charge transfer character of the TS structure of both species. This was conducted to evaluate whether the experimentally induced relative chemiexcitation yields could be explained by either CIEEL or CTIL mechanisms.

**Scheme 3:**
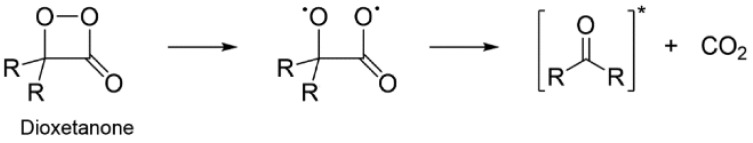
Schematic depiction of the thermolysis reaction of dioxetanone species, reproduced here with authorization from [62].

Previous theoretical research has shown that the thermolysis reaction of dioxetanones usually follows a stepwise pathway that consists of an initial peroxide O-O bond breaking, followed by C-C bond stretching (Scheme 3) [1,2,23,33,50,51,52,60,61,66,67]. This yields a chemiexcited ketone (here, F-CLMD) and CO_2_. The TS of these reactions is achieved by O-O bond breaking, which results in the formation of a biradical [1,2,23,33,50,51,52,60,61,66,67]. In the case of neutral species, the biradical tends to be formed by homolytic cleavage of the peroxide O-O bond, whereas for anionic species, it results from electron-transfer from an electron-rich group to the dioxetanone moiety [1,2,23,33,50,51,52,60,61,66,67].

Analysis of both neutral and anionic TS structures revealed <*S^2^*> values of ~1 (1.0 for neutral species and 0.9 for the anionic TS) for both species, which indicates that they possess biradical character. As indicated, this is consistent with existing literature [1,2,23,33,50,51,52,60,61,66,67]. For both species, the dioxetanone ring of the TS structure is associated with a broken peroxide O-O bond, but not with a cleaved C-C bond, which is in line with the typical stepwise biradical pathway associated with this type of compound [1,2,23,33,50,51,52,60,61,66,67]. 

**Figure 4:**
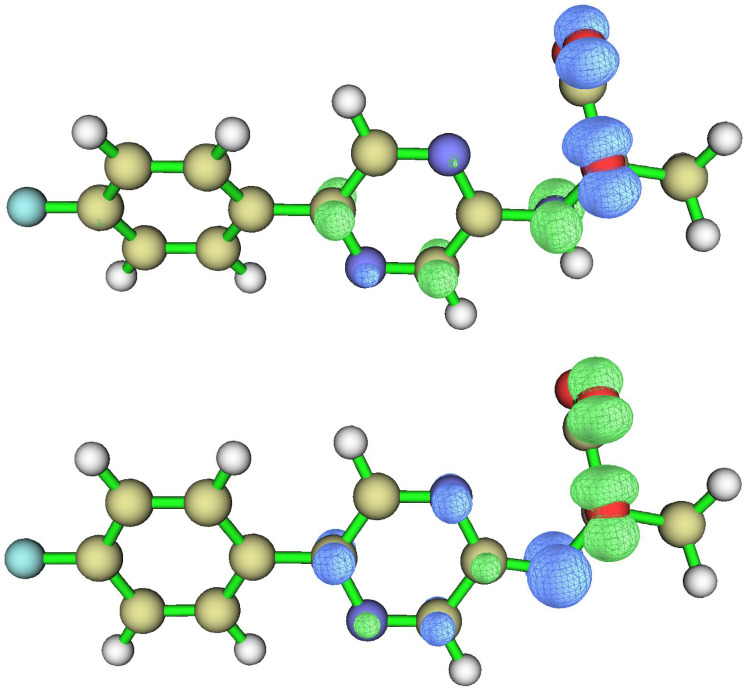
Electron spin density isosurfaces for neutral (top) and anionic (bottom) F-CLA dioxetanone.

To assess the electron transfer of both TS structures, we plotted their electron spin density isosurfaces (Figure 4). These were obtained using MultiWFN software, v. 3.8 [71], considering the calculations at the ωB97XD/6-31+G(d,p) level of theory (with Gaussian 09). The results obtained for the anionic species are consistent with existing literature regarding dioxetanones with this deprotonation state [1,2,23,33,50,51,52,60,61,66,67]. Namely, the electron spin density was delocalized between the two oxygen heteroatoms that constitute the peroxide O-O bond and the amidopyrazine moiety (with a focus on the deprotonated amide). This indicates that reaching the TS structure is associated with electron transfer between the amidopyrazine structure and the dioxetanone moiety, as is consistent with the CIEEL mechanism [1,2,23,33,50,51,52,60,61,66,67]. In fact, Saito and co-workers mentioned that the nitrogen anion could serve as a strong electron donor [48].

The analysis of possible charge transfer between amidopyrazine and dioxetanone was also performed by measuring the charge separation between moieties. This was achieved by calculating both the Hirshfeld atomic charges and the Voronoi deformation density atom population, which were obtained using MultiWFN software, v. 3.8 [71], considering the calculations at the ωB97XD/6-31+G(d,p) level of theory (with Gaussian 09). These results are presented in Table 1. Both charge analyses indicated that reaching the anionic TS structure is also associated with charge transfer from the amidopyrazine structure to the dioxetanone moiety, as is consistent with the CTIL mechanism and previous studies [1,2,24,34,51,52,53,61,62,67,68]. 

Given that anionic F-CLA dioxetanone is associated with both CIEEL/CTIL and more efficient singlet chemiexcitation, this could mean that one or both of these mechanisms could indeed explain the observed chemiexcitation, especially considering that the decomposition of neutral dioxetanones is typically associated with non-CIEEL/CTILL pathways [1,2,23,33,50,51,52,60,61,66,67]. That is, there is little charge separation between the moieties at the TS, and the homolytic cleavage of the peroxide O-O bond occurs instead of electron transfer to the dioxetanone moiety at the TS. However, this is not the case for neutral F-CLA dioxetanone. Interestingly, the decomposition of the neutral species is not the result of a homolytic peroxide O-O bond [1,2,23,33,50,51,52,60,61,66,67]. Instead, the electron spin density (Figure 4) indicates that the TS of this species is reached by electron transfer between the amidopyrazine and dioxetanones moieties, an indication of the involvement of the CIEEL pathway. Moreover, the electron spin density delocalization observed for neutral F-CLA dioxetanone was quite similar to what was seen for the corresponding anionic species (Figure 4).

Charge analysis, considering both Hirshfeld atomic charges and Voronoi deformation density, was also performed for neutral F-CLA dioxetanone (Table 1). The measurement of charge separation between the moieties revealed that this species also presents different behavior, in terms of charge transfer, than other neutral dioxetanones [1,2,23,33,50,51,52,60,61,66,67]. That is, we observed significant charge transfer from the amidopyrazine structure to the dioxetanone moiety, considering a relevant charge separation between the moieties, which is an indication of the involvement of the CTIL mechanism.

In short, the theoretical analysis indicated that the decomposition reaction of both neutral and anionic F-CLA dioxetanones should occur via a similar stepwise biradical pathway, with the TS being reached with electron/charge transfer from the amidopyrazine structure to the dioxetanone moiety. This signals the involvement of both CIEEL and CTIL mechanisms in the reactions of both species. Although the results regarding the anionic species are in line with existing literature [1,2,23,33,50,51,52,60,61,66,67], this is not quite the case for neutral dioxetanone. 

Considering all the obtained evidence, different conclusions can be reached. First, both species were found to undergo thermolysis via both CTIL and CIEEL mechanisms. Moreover, the behavior of these mechanisms was quite similar (electron/charge transfer between the amidopyrazine and dioxetanone moieties). Given this, it is difficult to ascertain what the actual differences between CTIL and CIEEL are, how/why they can be considered as independent mechanisms, and how one can explain behaviors that the other cannot. In fact, it should be remembered that the requirements of either electron-transfer-based or charge-transfer-based mechanisms are similar, and that charge transfer encompasses electron transfer [41,47]. Thus, it is not clear whether there is enough evidence to justify treating CIEEL and CTIL as two independent mechanisms. 

Furthermore, irrespective of their differences/similarities, both CIEEL and CTIL were developed to explain efficient chemiexcitation [40,41,45,46]. Thus, if they are valid regarding imidazopyrazinone CL/BL, it is expected that they could explain the differences in chemiexcitation efficiency. However, both neutral and anionic F-CLA dioxetanones were associated with both mechanisms, whereas the experimental results indicated that the chemiexcitation yield of the latter is higher than that of the former. Thus, neither CTIL nor CIEEL appears to explain the relative chemiexcitation efficiency of both species. In fact, the overall charge separation between moieties is significantly higher for neutral species than for anionic species (1.66*e* versus 0.73*e*). If these mechanisms were operative here, one could even expect that the species with the highest chemiexcitation yield would be the neutral species; however, the results indicate the opposite. Given this, the results indicate that neither CIEEL nor CTIL mechanisms explain the observed relative chemiexcitation yields, which is further evidence for the lack of applicability of these mechanisms for imidazopyrazinone-based systems. This is in line with the previously observed lack of correlation between charge/electron transfer and efficient chemiexcitation [33,48,49,50,51,52]. Therefore, alternative explanations for the chemiexcitation of imidazopyrazinone-based dioxetanones should be considered.

## 4. Conclusions

In this study, the CL reaction of a fluorinated F-CLA analog was investigated to gain insight into the possible mechanisms that control the chemiexcitation step of imidazopyrazinone-based CL and BL reactions. More specifically, the electron-donating hydroxyl group of native Coelenterazine was replaced by an electron-withdrawing fluorine (among other substitutions) to obtain more information regarding potential electron and/or charge transfer processes that may occur. The relevance of this study results from the fact that different researchers are using electron/charge-transfer-based mechanisms (CIEEL and CTIL) to explain chemiexcitation efficiencies, despite conflicting reports from the literature.

Luminometric analysis of the CL of this analog in aprotic media revealed significantly higher light-emission under basic conditions than under acidic conditions. Further analysis indicated that this higher light output was the result of the higher relative chemiexcitation efficiency of an anionic dioxetanone intermediate over the corresponding neutral species and not due to other factors. In fact, the results indicate that light output is higher under basic conditions despite lower fluorescent yields at that pH range, which further highlights the required higher chemiexcitation efficiency. 

Interestingly, theoretical calculations indicated that the decomposition of both anionic and neutral dioxetanones occurred with both electron and charge transfer, which signals the involvement of both CIEEL and CTIL mechanisms in the resulting chemiexcitation step. However, the involvement of both mechanisms in the chemiexcitation of species with different chemiexcitation yields puts in question their capacity to explain relative chemiexcitation efficiencies in this type of system. Furthermore, the occurrence of both mechanisms in the same reaction and in similar conditions also cast doubts on how truly differentiated and independent CTIL and CIEEL really are. 

In conclusion, this study was able to obtain further insight into the potential mechanisms underlying the chemiexcitation step of imidazopyrazinone-based CL/BL systems, while providing evidence that indicates that the pursuit of alternative explanations for the chemiexcitation of imidazopyrazinone-based dioxetanones should be considered.

## 5. Patents

WO20219211808—Chemiluminescent Imidazopyrazinone-Based Photosensitizers with Available Singlet and Triplet Excited States.

## Figures and Tables

**Table 1:** Charge separation between the amidopyrazine and dioxetanone moieties of both neutral and anionic species of F-CLA dioxetanone. Charge separation was measured by calculating both the Hirshfeld atomic charges and the Voronoi deformation density atom population, which were obtained using MultiWFN software, v. 3.8 [71], considering calculations at the ωB97XD/6-31+G(d,p) level of theory (with Gaussian 09).

**Neutral F-CLA Dioxetanone**		
	**Hirshfeld Charges**	**Voronoi Deformation Density**
Amidopyrazine	0.79	0.83
Dioxetanone	−0.79	−0.83
**Anionic F-CLA Dioxetanone**		
	**Hirshfeld Charges**	**Voronoi Deformation Density**
Amidopyrazine	−0.16	−0.14
Dioxetanone	−0.84	−0.86

## Data Availability

Not applicable.
